# Osteoblastoma of the Capitate Bone: Case Report

**DOI:** 10.1055/s-0041-1724084

**Published:** 2021-04-19

**Authors:** Lucas Gonçalves Dias de Lima, Ubiratan Brum de Castro, Gustavo Pacheco Ferreira Martins

**Affiliations:** 1 Departamento de Ortopedia e Traumatologia, Hospital das Clínicas, Universidade Federal de Minas Gerais, Belo Horizonte, MG, Brasil; 2 Faculdade de Medicina, Universidade Federal de Minas Gerais, Belo Horizonte, MG, Brasil; 3 Hospital das Clínicas, Universidade Federal de Minas Gerais, Belo Horizonte, MG, Brasil

**Keywords:** carpal bones, osteoblastoma, bone neoplasms, hand

## Abstract

Carpal bone tumors must be investigated in clinical cases of chronic wrist pain with no previous trauma. Intraosseous ganglion, enchondroma, osteoid osteoma, and, less commonly, osteoblastoma are potential causes of osteolytic lesions affecting the carpal bones. In most cases, the clinical presentation alone is not enough to differentiate such lesions. Knowledge of certain characteristics, including the radiological and histopathological aspects of each of these tumors, is critical in order to make the differential diagnosis. We present a rare case of osteoblastoma of the capitate bone and review the literature on the subject.

## Introduction

Carpal bone tumors are suspected in clinical presentations of chronic wrist pain with no history of previous trauma. Injuries with osteolytic features are often intraosseous ganglia, enchondroma, osteoid osteoma, and, less commonly, osteoblastoma.

Osteoblastomas are benign neoplasms that form bone cells. They represent 1% of tumors, and their most common locations are the posterior elements of the spine, pelvis, and long bones. The carpal bones are rarely affected, and involvement of the capitate bone is even less common. 1 2 3 The present study describes a case of osteoblastoma of the capitate bone and reviews the current literature on the subject.

## Case Report

A 40-year-old, female, right-handed patient with a history of progressive pain on the dorsal face of the right hand for 2 years. The patient denied previous traumas, infections or other symptoms associated with the condition.

The patient presented a swelling at the affected area and functional impairment to perform basic activities of daily living.

An examination revealed a slight reduction in flexion in the right wrist, decreased grip strength, and pain on palpation of the capitate bone. The ectoscopy showed no changes.

**Fig. 1:**
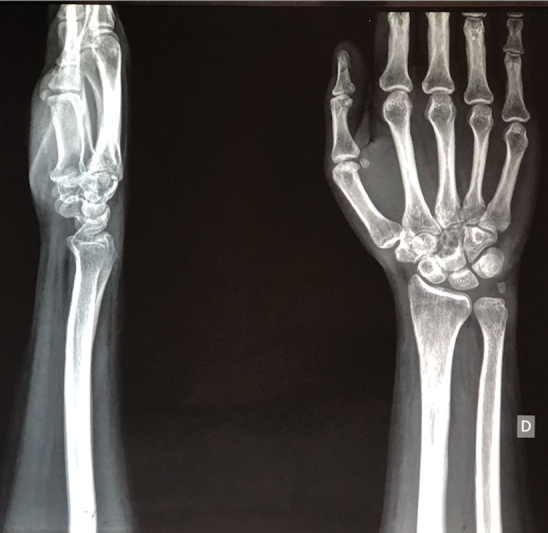
Initial radiograph of the right wrist, revealing a well-defined osteolytic lesion at the capitate bone.

Initial radiographs showed a well-defined osteolytic lesion with a sclerotic margin and a lobulated aspect at the capitate bone ( Fig. 1 ).

**Fig. 2:**
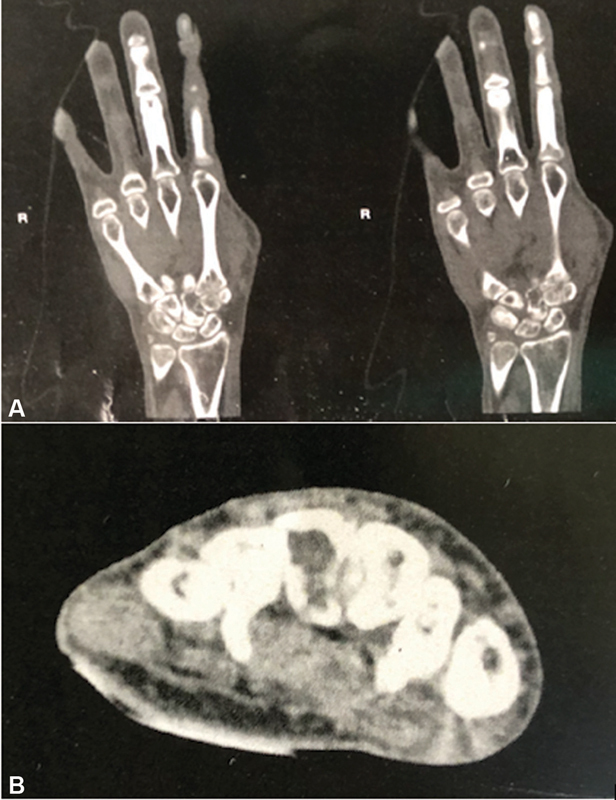
Computed tomography of the right hand. Coronal ( **A** ) and axial ( **B** ) sections. There is a well-defined hypodense lesion at the right capitate bone, with hyperdense margins, and no involvement of other hand bones.

There were no calcifications or extensions to soft parts or other bones. Due to the non-specific aspect on radiography, a computed tomography was requested, revealing a lesion with a central hypodense component and well-defined hyperdense margins restricted to the distal portion of the capitate bone ( Fig. 2 ).

**Fig. 3:**
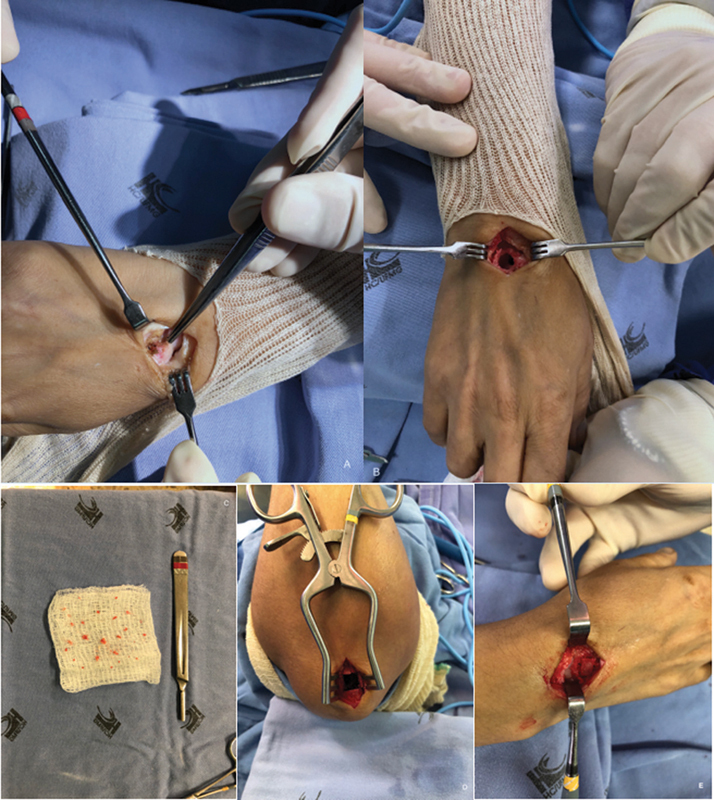
Perioperative period. ( **A** ) Perioperative aspect of the lesion at the right capitate bone. ( **B** ) Bone cavity after lesion curettage. ( **C** ) Resected specimen sent for an anatomopathological examination. ( **D** ) Bone-graft donor area at the right olecranon. ( **E** ) Bone defect filled with an olecranon graft.

Surgical treatment with intralesional resection and an ipsilateral olecranon bone graft was indicated because of the significant pain and functional impairment. The resected specimen was sent for an anatomopathological study ( Fig. 3 ).

**Fig. 4:**
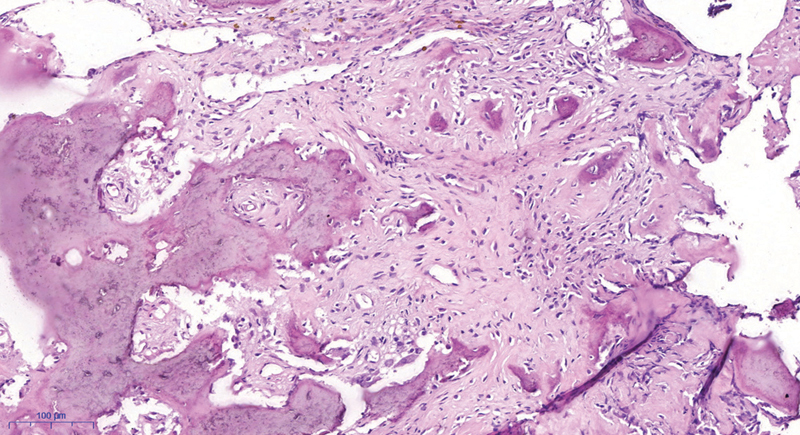
Histopathological aspect of the resected specimen, revealing young bone tissue and some trabeculae of mature bone tissue, with no atypia, permeated by fibrous tissue.

The histopathological analysis of the bone-tissue fragments revealed a benign lesion consisting of young and mature bone tissue, with no atypia, permeated by fibrous tissue, diagnosed as an osteoblastoma ( Fig. 4 ).

**Fig. 5:**
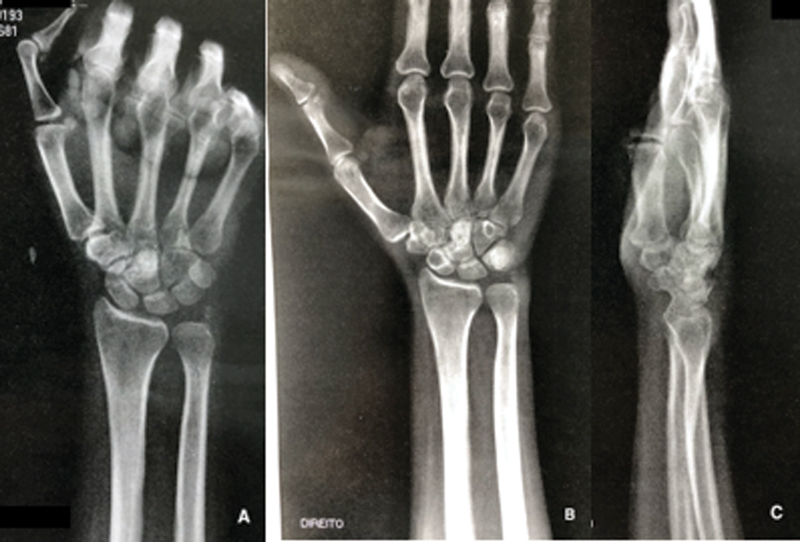
Radiographs of the right wrist eight weeks after surgery. Good integration of the bone graft into the right capitate bone, with no signs of lesion recurrence. Posteroanterior view with clenched fist ( **A** ), posteroanterior view ( **B** ), and lateral view ( **C** ) of the right wrist.

One week after the procedure, the patient presented a significant improvement in pain, which was sustained at subsequent evaluations at 3, 6 and 12 weeks after the procedure ( Fig. 5 ).

The patient showed good functional recovery according to the Disability of the Arm Shoulder and Hand (DASH) questionnaire, and gradually resumed her activities.

## Discussion

Reports of carpal bone osteoblastoma are infrequent, and the involvement of the capitate bone is even rarer. Kaptan and Atmaka 2 and Afshar 3 reported this tumor location in their respective studies, while Murray et al., 4 in their series with 26,800 primary bone tumors, cite a single case of osteoblastoma of the capitate bone.

Osteoblastomas are benign neoplasms arising from bone tissue. Microscopically, they are characterized by trabecular bone and fibrovascular stroma with the production of primitive osteoid tissue. 1 5

These tumors affect mainly patients in their second and third decades of life, preferably males, in a 2-3:1 ratio. 1 2 3 Our patient, a 40-year-old woman, was did not fit the most common epidemiological profile.

Although they can affect any bone, 40% to 50% of the lesions are at the spine, preferably its posterior elements. 1 They rarely affect hand bones. 2 3 As such, the diagnosis of osteoblastoma is often not considered in cases of primary carpal bone tumors.

Radiographically, osteoblastoma presents as a centralized, mineralized nidus with a surrounding radiolucent halo and reactive sclerosis, or as a well-defined, mixed (both lytic and blastic) mass with sclerotic margins. 1 5 Computed tomography and magnetic resonance imaging reveal a heterogeneous, circumscribed mass, with cystic components, edema, and reactive bone. 5 Progressive, constant pain unresponsive to salicylates is the most common symptom, 6 as in our case.

Osteolytic lesions are not a frequent cause of chronic wrist pain, 6 but they must be considered, especially when there is no history of local trauma. Some examples are intraosseous ganglion, osteoid osteoma, enchondroma and osteoblastoma. 6 7 Knowledge of the clinical characteristics of each of these lesions is essential to differentiate them.

The differentiation of osteoblastomas and osteoid osteomas is a frequent challenge. Osteoid osteomas are more frequent in the hand. 8 It typically presents as a lesion < 1.5 cm, with a central nidus surrounded by a sclerotic zone. The pain is severe, worse at night, and relieved by salicylates. 5

Microscopically, both lesions are very similar; bone trabeculae lined by a single layer of osteoblasts, a tapered circumscription, and a loose arrangement of tissue are the characteristics that favor the diagnosis of osteoblastoma. 1

In bone tumors of the hand, enchondromas are an important differential diagnosis, since they are the most common primary bone tumor of the hand. 6 8 Their classic radiographic appearance is that of a a well-defined osteolytic lesion with dotted calcifications. 6

Another differential diagnosis, the intraosseous ganglion is an asymptomatic, benign cystic lesion, and an incidental finding on imaging studies in approximately 80% of the cases. 9

Radiographically, it presents as a cystic, unilocular, non-expansive, well-defined lesion with sclerotic borders. Magnetic resonance imaging shows a hyposignal lesion in T1-weighted images with hypersignal in fat-suppressed sequences. Microscopically, it is characterized by mucoid tissue, myxomatous degeneration of connective tissue, and fibrous walls. 7

The treatment of osteoblastomas is still a matter of debate. Studies suggest that the tumor may undergo malignant transformation, 7 and recurrence rates between 10% and 20% are reported. 3 5 Therefore, some authors indicate a range of procedures, from complete lesion resection to curettage with or without bone grafting. 3 10 Castelló et al. 10 described good outcomes after curettage and defect filling with a bone graft.

In the case herein reported, the clinical presentation and radiographic appearance initially did not enable a specific diagnosis of an osteolytic lesion. However, due to the non-aggressive behavior of the lesion, its benign characteristics, and the lack of extension to soft parts or other bones on imaging tests, we decided for curettage and defect filling with a bone graft. The resected specimen was sent for an anatomopathological examination, which later suggested the diagnosis of an osteoblastoma.

We presented a rare case of osteoblastoma of the capitate bone as a cause of chronic wrist pain, in addition to a review on the subject. Thus, osteoblastomas can be considered a diagnosis of exclusion for an osteolytic lesion at the level of the carpal bone.
