# Supplementary material for: Osteoblastoma of the Capitate Bone: Case Report
Source: Rev Bras Ortop (Sao Paulo). 2021 Apr 19;59(2):e327–30. doi: 10.1055/s-0041-1724084 (PMC11006508; doi:10.1055/s-0041-1724084)
Supplement: Related texts

# Research article: Osteoblastoma do capitato: Relato de caso

**Keywords:** ossos do carpo, osteoblastoma, neoplasias ósseas, mão

## Abstract

Os tumores dos ossos do carpo devem ser investigados em quadros clínicos de dor crônica no punho sem trauma prévio. Gânglio intraósseo, encondroma, osteoma osteoide, e, menos comumente, osteoblastoma são possíveis causas de lesões osteolíticas que afetam os ossos carpais. Na maioria dos casos, a apresentação clínica por si só não é suficiente para diferenciar essas lesões. Conhecer características como o aspecto radiológico e histopatológico de cada uma delas é parte fundamental do diagnóstico diferencial. Apresentamos um caso raro de osteoblastoma do osso capitato, e também fazemos uma revisão da literatura sobre o assunto.

## Introdução

Os tumores ósseos do carpo devem ser pesquisados diante de um quadro clínico de dor crônica do punho sem histórico de trauma prévio. Quando apresentam características osteolíticas, as causas mais comuns são os gânglios intraósseos, encondroma, osteoma osteóide e, menos comumente, osteoblastoma.

Osteoblastomas são neoplasias benignas formadoras de células ósseas. Representam 1% dos tumores, e suas localizações mais comuns são os elementos posteriores da coluna vertebral, pelve, e ossos longos. Raramente os ossos do carpo são acometidos, sendo o envolvimento do capitato ainda menos comum. 1 2 3 Neste estudo, descreve-se um caso de osteoblastoma do capitato, assim como se faz uma revisão da literatura sobre o tema.

## Relato de Caso

Paciente de 40 anos, destra, com histórico de dor na face dorsal da mão direita, progressiva, com 2 anos de evolução. Negava traumas prévios, infecções, ou outros sintomas associados ao quadro.

Apresentou-se com aumento de volume da área afetada e prejuízo funcional para a realização de atividades básicas de vida diária.

Ao exame, encontrava-se com a flexão do punho direito discretamente limitada, diminuição da força de preensão, e dor à palpação do capitato. A ectoscopia não demonstrava alterações.

As radiografias iniciais mostraram uma lesão osteolítica, bem definida, com margem esclerótica e aspecto lobulado no capitato ( Fig. 1 ).

**Fig. 1:** Radiografia inicial do punho direito. Observa-se presença de lesão osteolítica bem definida no osso capitato.

Não foram vistas calcificações ou extensão para partes moles ou outros ossos. Devido ao aspecto inespecífico à radiografia, foi solicitada tomografia computadorizada, que apresentou lesão com componente hipodenso central e margens hiperdensas, bem definidas, restrita à porção distal do capitato ( Fig. 2 ).

**Fig. 2:** Tomografia computadorizada da mão direita. Cortes coronal ( **A** ) e axial ( **B** ). Nota-se presença de lesão hipodensa em capitato direito, bem definida, com margens hiperdensas, sem acometimento de outros óssos da mão.

Em função do quadro álgico significativo e comprometimento funcional, foi indicado tratamento cirúrgico com ressecção intralesional e preenchimento com enxerto ósseo de olécrano ipsilateral. O material curetado foi enviado para estudo anatomopatológico ( Fig. 3 ).

**Fig. 3:** Peroperatório. ( **A** ) Aspecto peroperatório da lesão em capitato direito. ( **B** ) Cavidade óssea após curetagem da lesão. ( **C** ) Material curetado enviado para exame anatomopatológico. ( **D** ) Área doadora de enxerto ósseo do olecrano direito. ( **E** ) Cavidade óssea preenchida por enxerto de olecrano.

A análise dos cortes histológicos dos fragmentos de tecido ósseo mostrou se tratar de lesão benigna composta por tecido ósseo jovem e maduro, sem atipias, permeada por tecido fibroso, sendo diagnosticada como osteoblastoma ( Fig. 4 ).

**Fig. 4:** Aspecto histopatológico do material enviado para análise. Observa-se tecido ósseo jovem e algumas trabéculas de tecido ósseo maduro, sem atipias, permeados por tecido fibroso.

A avaliação clínica da paciente 1 semana após o procedimento revelou melhora significativa do quadro álgico, o que se manteve nas avaliações posteriores com 3, 6 e 12 semanas de pós-operatório ( Fig. 5 ).

**Fig. 5:** Radiografias do punho direito com oito semanas de pós-operatório. Observa-se boa integração do enxerto ósseo ao capitato direito, sem sinais de recidiva da lesão. Radiografia posteroanterior com o punho cerrado ( **A** ), posteroanterior ( **B** ) e de perfil ( **C** ) do punho direito.

A paciente apresentou boa recuperação funcional segundo os parâmetros do questionário Disabilities of the Arm, Shoulder and Hand (DASH, Disfunções do Braço, Ombro e Mão), e retornou gradualmente às suas atividades.

## Discussão

Relatos de osteoblastoma que afeta os ossos do carpo são infrequentes, sendo o acometimento do capitato ainda mais raro. Kaptan e Atmaka 2 e Afshar 3 apresentaram relato dessa localização tumoral em seus respectivos trabalhos, ao passo que Murray et al., 4 em sua série de 26.800 tumores ósseos primários, citam apenas 1 caso de osteoblastoma do capitato.

Osteoblastomas são neoplasias benignas formadoras de tecido ósseo. Microscopicamente, caracterizam-se por osso trabecular e estroma fibrovascular com produção de tecido osteoide primitivo. 1 5

Eles afetam principalmente pacientes na segunda e terceira décadas de vida, preferencialmente do sexo masculino (na razão de 2-3:1). 1 2 3 No caso apresentado, a paciente estava fora do padrão epidemiológico mais comum, tratando-se de uma mulher de 40 anos.

Os osteoblastomas podem acometer qualquer osso; porém, entre 40% e 50% das lesões localizam-se na coluna vertebral, preferencialmente nos elementos posteriores. 1 Raramente acometem os ossos da mão. 2 3 Por essas características, o diagnóstico de osteoblastoma não é frequentemente considerado em casos de tumores ósseos primários do carpo.

Radiograficamente, o osteoblastoma caracteriza-se como nicho central mineralizado com halo radiolúcido circundante e esclerose reativa, ou como massa bem definida, mista (lítica e blástica), com margens escleróticas. 1 5 Tomografia computadorizada e ressonância magnética podem mostrar massa circunscrita, heterogênea, que apresenta componentes císticos, edema e osso reativo. 5 Dor progressiva, constante, sem resposta a salicilatos é o sintoma mais comum, 6 como observado no caso descrito.

As lesões osteolíticas não são causa frequente de dor crônica no punho, 6 mas devem ser pensadas principalmente na ausência de histórico de trauma local. Alguns exemplos são o gânglio intraósseo, o osteoma osteoide, o encondroma, e o osteoblastoma. 6 7 Conhecer as características clínicas de cada uma dessas lesões é fundamental para diferenciá-las.

Uma dúvida frequente é a diferenciação entre osteoblastoma e osteoma osteoide. Na mão, o osteoma osteoide é mais frequente. 8 Classicamente, apresenta-se como lesão < 1,5cm, com *nidus* central circundado por zona de esclerose. A dor é intensa, pior à noite, com alívio importante com o uso de salicilatos. 5

Microscopicamente, as lesões são muito semelhantes, sendo o revestimento das trabéculas ósseas por uma camada única de osteoblastos, a circunscrição afilada, e o arranjo solto do tecido características que favorecem o diagnóstico de osteoblastoma. 1

No caso dos tumores que envolvem os ossos da mão, o encondroma é diagnóstico diferencial importante, uma vez que se trata do tumor primário ósseo mais comum das mãos. 6 8 Sua aparência radiográfica clássica é a de lesão osteolítica bem definida, com calcificações pontilhadas. 6

Outro diagnóstico diferencial, o gânglio intraósseo é uma lesão cística benigna, sendo assintomática e um achado incidental em exames de imagem em aproximadamente 80% dos casos. 9

Na radiografia, apresenta-se como lesão cística, unilocular, não expansiva, bem definida, com bordas escleróticas. A ressonância magnética mostra lesão de hipossinal em T1 e hipersinal nas sequências com supressão de gordura. À microscopia, o gânglio intraósseo é caracterizado por tecido mucoide, degeneração mixomatosa de tecido conjuntivo e paredes fibrosas. 7

O tratamento dos osteoblastomas ainda é motivo de debates. Estudos sugerem que o tumor pode sofrer transformação maligna, 7 além de taxas de recorrência entre 10% e 20%. 3 5 Sendo assim, há autores que indicam desde ressecção completa da lesão até curetagem associada ou não a enxertia óssea. 3 10 Castelló et al. 10 descreveram bons resultados após curetagem seguida de preenchimento com enxerto ósseo.

No caso relatado, inicialmente, o quadro clínico e a aparência radiográfica não permitiram um diagnóstico específico da lesão osteolítica. Contudo, em função do comportamento não agressivo da lesão, suas características benignas, e a ausência de extensão para partes moles ou outros ossos aos exames de imagem, optou-se pela realização de curetagem com preenchimento com enxerto ósseo, sendo o material coletado enviado para exame anatomopatológico, que sugeriu posteriormente o diagnóstico de osteoblastoma.

Apresentou-se um caso raro de osteoblastoma do capitato como causa de dor crônica do punho, assim como foi feita uma revisão e atualização do tema. Dessa forma, o osteoblastoma pode ser considerado um diagnóstico de exclusão quando nos deparamos com uma lesão osteolítica no nível do carpo.
